# Retraction Note: Detection of *Helicobacter* spp. DNA in the colonic biopsies of stray dogs: molecular and histopathological investigations

**DOI:** 10.1186/s13000-016-0572-5

**Published:** 2016-11-02

**Authors:** Fatemeh Soghra Abdi, Shahram Jamshidi, Farhad Moosakhani, Farhang Sasani

**Affiliations:** 1 Department of Small Animal Internal Medicine, Resident of Specialized Veterinary Sciences, Science and Research Branch, Islamic Azad University (IAU), NO.14, Corner of Parvaz 1, Payam Blvd., Saadat Abad, Tehran Iran; 2 Department of Clinical Sciences, Faculty of Veterinary Medicine, University of Tehran, Tehran, Iran; 3 Department of Microbiology, Faculty of Veterinary Medicine, University of Karaj, Karaj, Iran; 4 Department of Pathology, Faculty of Veterinary Medicine, University of Tehran, Tehran, Iran

## Retraction

The Editor-in-Chief and Publisher have retracted this article [1] because the scientific integrity of the content cannot be guaranteed. An investigation by the Publisher found it to be one of a group of articles we have identified as showing evidence suggestive of attempts to subvert the peer review and publication system to inappropriately obtain or allocate authorship. This article showed evidence of plagiarism (most notably from the article cited [2]) and peer review and authorship manipulation.
